# Green County Medical Society

**Published:** 1840-11

**Authors:** 


### GREEN COUNTY MEDICAL SOCIETY.

The physicians of Green county, Ohio, in 1839, formed themselves into a society under this style. The officers for the present year are Dr. Matthias Winans, President; Dr. Joshua Martin, Vice President; Dr. J. J. M'Illhenny, Recording Secretary; Dr. John Dawson, Corresponding Secretary; Dr. Joseph Templeton, Librarian; Dr. James Cummings, Treasurer.

At each meeting a person is appointed to read a dissertation at the next; and it is expected that every member of the society, will report all the important cases which occur in his practice. These reports may be made either in writing or orally; and are liable to critical examination. They are, indeed, themes for discussion; and exercises thus gotten up, cannot fail to be eminently useful.

The Society has adopted a system of rules and regulations for the government of its members in their professional intercourse with one another, and also a fee bill, designed to produce uniformity of deportment and charging.

Should Societies of this kind be organized in all the counties of the West, their influence would prove salutary in the highest degree to the character and interests of the profession.

The Green County Society holds its meetings in the town of Xenia. D.

